# Causal relationships between blood lipids and major psychiatric disorders: Univariable and multivariable mendelian randomization analysis

**DOI:** 10.1186/s12920-023-01692-8

**Published:** 2023-10-18

**Authors:** Bozhi Li, Yue Qu, Zhixin Fan, Xiayu Gong, Hanfang Xu, Lili Wu, Can Yan

**Affiliations:** Integrative Medicine Research Center, School of Basic Medical Sciences, Guangzhou University of Chinese Medicine, Guangzhou, Guangdong China

**Keywords:** Blood lipids, Depression, Anxiety, Panic, Post-traumatic stress disorder, Alzheimer’s disease, Schizophrenia, Mendelian randomization analysis

## Abstract

**Background:**

Whether the positive associations of blood lipids with psychiatric disorders are causal is uncertain. We conducted this two-sample Mendelian randomization (MR) analysis to comprehensively investigate associations of blood lipids with psychiatric disorders.

**Methods:**

Univariable and multivariable models were established for MR analyses. Inverse variance-weighted (IVW) MR was employed as the main approach; weighted median and MR-Egger were used as sensitivity analysis methods. The possibility of violating MR assumptions was evaluated utilizing several sensitivity analyses, including heterogeneity statistics, horizontal pleiotropy statistics, single SNP analysis, leave-one-out analysis and MR-PRESSO analysis. As instrumental variables, we screened 362 independent single-nucleotide polymorphisms (SNP) related to blood lipids from a recent genome-wide association study involving 76,627 individuals of European ancestry, with a genome-wide significance level of p < 5 × 10^− 8^. Summary-level information for the six psychiatric disorders was extracted from Psychiatric Genomics Consortium and Alzheimer Disease Genetics Consortium.

**Results:**

We observed eight significant associations in univariable MR analysis, four of which were corroborated by multivariable MR (MVMR) analysis modified for the other three lipid traits: high-density lipoprotein cholesterol (HDL-C) level with the risk of PTSD (OR = 0.91, 95% CI = 0.85–0.97, *p* = 0.002) and AD (OR = 0.79, 95% CI = 0.71–0.88, *p* < 0.001) and triglycerides (TG) level with the risk of MDD (OR = 1.02, 95% CI = 1.003–1.03, *p* = 0.01) and panic disorder (OR = 0.83, 95% CI = 0.74–0.92, *p* < 0.001). In addition, four associations were not significant in MVMR analysis after adjustment for three lipid traits: total cholesterol (TC) level with the risk of PTSD, low-density lipoprotein cholesterol (LDL-C) level with the risk of MDD and AD and TG level with the risk of AD.

**Conclusions:**

Our results show that blood lipids and psychiatric disorders may be related in a causal manner. This shows that abnormal blood lipid levels may act as reliable biomarker of psychiatric disorders and as suitable targets for their prevention and treatment.

**Supplementary Information:**

The online version contains supplementary material available at 10.1186/s12920-023-01692-8.

## Introduction

Recently, psychiatric diseases have emerged as a major worldwide public health issue with substantial morbidity and mortality [1], impacting more than a quarter of the global population [2]. However, the pathogenesis of psychiatric diseases is still unknown due to their complex etiology, and there are relatively limited therapies that are both effective and long-lasting [3]. In order to help with the creation of new preventative and intervention measures, a more comprehensive knowledge of the pathophysiology and possible risk factors of psychiatric diseases is highly required.

The cause of psychiatric diseases is still unknown to date. There have been considerable efforts to explore biological biomarkers related to and/or causing psychiatric disorders, with blood lipid being a potential etiological variable that has been quite extensively investigated. For example, previous studies indicated that high triglycerides (TG) quantity was connected to a high risk of major depressive disorder (MDD) [4], anxiety disorder [5], posttraumatic stress disorder (PTSD) [6], Alzheimer’s disease [7] and Schizophrenia [8]. However, other studies failed to prove such relationships [4, 9–11]. Therefore, these findings are inconclusive, indicating positive, inverse or non-significant associations among blood lipids and psychiatric disorders. In general, confounders or selection biases inherent in traditional observational studies may, to some degree, influence the link between blood lipids and psychiatric diseases. Furthermore, it is still debatable whether dyslipidemia is a root cause or a consequence of psychiatric issues. As a result, verification is still needed for any probable underlying cause-effect association.

In an attempt to resolve these discrepancies, Mendelian randomization (MR), a genetic epidemiological method has been applied in recent studies to define whether genetically determined blood lipid levels are associated with the risk of psychiatric disorders. A correctly performed MR may complement observational research and promote triangulation of evidence by using genetic differences, including single nucleotide polymorphisms (SNPs), as instrumental variables (IVs) for adjustable disease risk factors or exposures [12]. This is due to genetic variations being randomly distributed during gamete formation, independently assorted during meiosis, and have no influence on susceptibility to the disease, which minimize the likelihood of inverse causation and confounding [13, 14].

Only a few studies have examined the relationship between blood lipids and the risk of MDD, Alzheimer’s disease, and schizophrenia in earlier MR research [15–19]; however, these studies did not evaluate the relationship between blood lipids and the risk of anxiety disorders, panic disorders, or PTSD. Concerning the probable causative significance of lipid profiles in psychiatric conditions, several MR investigations have revealed conflicting results [15–17]. Considering that several lipids are associated and pleiotropic, many of these investigations also failed to account for numerous lipid profiles, which may have restricted their results [20]. To get more clear findings, further investigation is required. Here, we conducted a two-sample bidirectional MR investigation utilizing recent genome-wide association studies (GWASs) and a summary-level two-sample univariable and multivariable MR framework to ascertain whether levels of lipid profiles are associated with psychiatric illnesses and to assess the direction of such correlations.

## Methods

### Study design

**Fig. 1:**
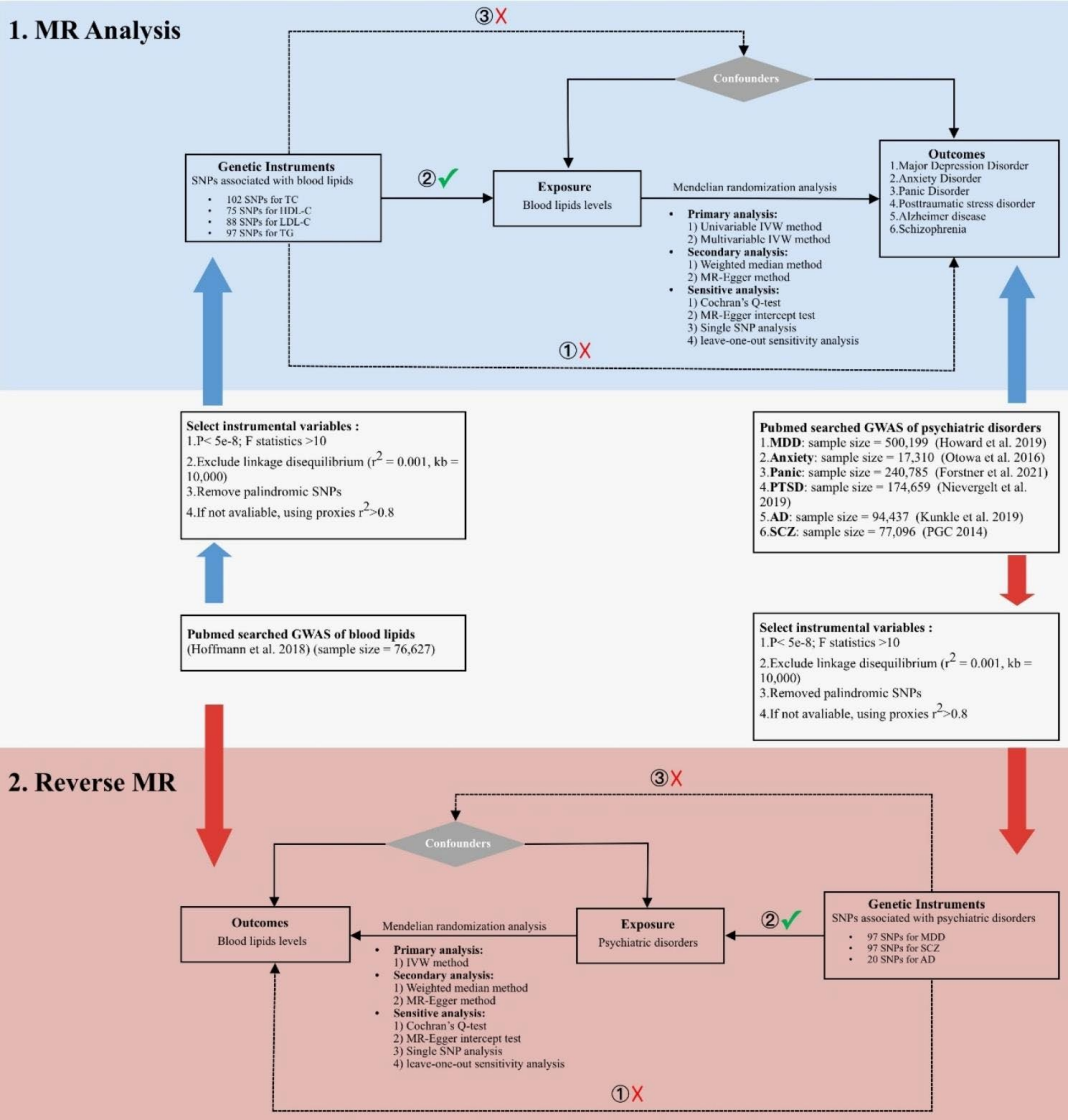
Bidirectional Mendelian randomization study workflow. Abbreviations: LDL-C, Low density lipoprotein cholesterol; HDL-C, High density lipoprotein cholesterol; TG, Triglycerides; TC, Total cholesterol; MR, Mendelian randomization; IVW, inverse-variance weighted MR; SNP, single nucleotide polymorphism

In this investigation, we employed a two-sample MR method to evaluate the causal relationship between blood lipid profile and psychiatric disorders (MDD, anxiety disorder, panic disorder, PTSD, Alzheimer’s disease (AD), and schizophrenia). Three major assumptions served as the foundation for this MR analysis: [1] Genetic variants employed as instrumental variables should be strongly correlated with lipid profile; [2] genetic variants utilized as instrumental variables should not be correlated with any confounding factors of the relationship among both lipid profile and psychiatric illness; and [3] genetic variants should only influence the risk of the psychiatric conditions via blood lipids (Fig. 1). We included publicly available summary statistics from seven predominantly European ancestry GWAS sources (Supplementary Table 1). No institutional review board authorization was needed for this study’s ethical conduct since all evaluations presented were based on publically accessible summary data.

### Blood lipids data

A large electronic health record-based genome-wide investigation of blood lipids in the multi-ethnic Genetic Epidemiology Resource on Adult Health and Aging (GERA) cohort (n = 94,674 patients with untreated lipid values) provided genome-wide association data for HDL, LDL, TG, and TC [21]. Over 20 million SNPs and 478,866 longitudinal, untreated blood lipid readings from electronic health records are available in this resource. It’s crucial that genetic variations substantially predict the exposure when conducting an MR study. The longitudinal data derived from electronic health records (EHRs) for use in this GERA GWAS has been revealed to diminish phenotypic variance, elevating statistical power and variant discovery [22, 23], which improves heritability estimation. Indicating substantial genetic modulation of the lipid profiles employed as exposures, the heritability estimates for all the lipid traits depending on the GCTA approach [24] were 32.8%, 23.3%, 30.5%, and 25.3% for high density lipoprotein cholesterol (HDL-C), low density lipoprotein cholesterol (LDL-C), TG, and total cholesterol (TC). Only people (N = 76,627) with European ancestry were included for the study’s purposes and to match with the GWAS for psychiatric disease. Supplementary Table 1 shows further details on the data source.

### Psychiatric disorders data

The most recent GWAS summary data for the six psychiatric illnesses were acquired from the Alzheimer Disease Genetics Consortium (ADGC) and the Psychiatric Genomics Consortium (PGC) (https://www.med.unc.edu/pgc/download-results/) (ADGC). The PGC is the largest and most systematic genomics effort in the history of psychiatry, containing a wealth of significant outcomes about the genetic architecture of psychiatric illnesses [25]. Utilizing genetic data from the European population based on exposure data helped to eliminate possible bias caused by population heterogeneity. The following were the respective sample sizes: 170,756 cases and 329,443 controls for MDD [26], 7016 cases and 14,745 controls for anxiety disorder [27], 2408 cases and 228,470 controls for panic disorder [28], 32,428 cases and 174,227 controls for PTSD [29], 21,982 cases and 41,944 controls for AD [30] and 36,989 cases and 113,075 controls for SCZ [31]. Detailed information on the data source is provided in Table S1. The primary investigations of each disease included details on genetic association analysis, quality control, genotyping, and imputation.

### Instrumental variable selection

We obtained genome-wide association data of lipid profiles from the multi-ethnic GERA cohort, including 76,627 samples of European descent and over 20 million SNPs (Supplementary Table 1). SNPs eligible for IVs were chosen from the exposure data using five quality control processes. First, SNPs that matched genome-wide significance (*p* < 5 × 10^− 8^) were selected for each trait. Second, the chosen SNPs also had to be independent of one another, so clumping was conducted (criteria: r^2^ = 0.001, kb = 10,000) to rule out linkage disequilibrium (LD) between them. Third, we harmonized exposure and outcome to confirm that these have the same effect allele. Fourth, we eliminated palindromic SNPs with intermediate allele frequencies to prevent alteration of strand orientation or allele coding. Finally, we assessed the F parameter to examine instrument strength. An F statistic was calculated utilizing the following formula: F = R^2^ × (n − 2) / (1 − R^2^), where R^2^ is the proportion of variance in instruments based on the formula R^2^ = 2 × effect allele frequency × (1 − effect allele frequency) × (Beta / standard deviation (SD, equals 1)^2^ and n represents the sample size. F > 10 revealed a reduced risk of weak instrument bias. A Phenoscanner analysis was applied to the instrumental variables in order to identify any known risk factors for psychiatric disorders that might confound our analysis (Supplementary File S2 B). Any SNPs that were associated with these confounders were then excluded from our study. Ultimately, 362 lipid related SNPs (102 for TC, 75 for HDL-C, 88 for LDL-C, and 97 for TG) were selected for final analysis. The details of the instrumental variables can be found in Supplementary File S2 A.

### Sample independence

Weak instrument bias in MR investigations may be increased by participant overlap in samples used to determine genetic correlations between exposures and results. In our analysis, we excluded this overlap. The summary statistics of blood lipids and psychiatric disorders were from completely different datasets.

### Univariable mendelian randomization

We employed inverse variance-weighted (IVW) MR as the main technique for univariable MR analysis. Nevertheless, we utilized supplementary MR-Egger and weighted median-based regression approaches, which include alternative IV assumptions, for sensitivity analysis to enhance the robustness of our findings since IVW only provides consistent estimates when all genetic variations are valid IVs. With at least 50% of the weight in the analysis coming from reliable instrumental variables, the weighted median analysis may provide estimates that are consistent [32]. Even when all genetic variations are invalid IVs, MR-Egger regression may provide consistent estimates. However, regarding accuracy, IVW estimates are more reliable than MR-Egger and Weighted Median estimates. Therefore, we mainly took IVW estimates as the result and supplemented them with the MR-Egger and Weighted Median estimates. The odds ratios (ORs) used to represent the MR estimations indicate how a one-unit increase in exposures (blood lipids) affects the risk of outcomes (psychiatric disorders). For multiple comparisons, the Benjamini & Hochberg adjustment was used. A suggestive connection between the exposure and the result was defined as a p-value < 0.05 but Benjamini-Hochberg adjusted p-value > 0.05.

We performed various tests to evaluate potential IV violations. First, the fixed-effect IVW method’s heterogeneity across various IVs was assessed employing Cochran’s Q-test, which also supplied useful information into pleiotropy. Significant variability in the Wald ratios for each IV in MR assessment utilizing several IVs might reveal a variety of possible issues, notable among which is that at least one (but perhaps many or even all) SNP is displaying horizontal pleiotropy. A multiplicative random-effects IVW model would be used if the heterogeneity is substantial. Second, the pleiotropy of IVs was assessed using the MR-Egger intercept analysis. It was considered that a zero MR-Egger intercept (p > 0.05) demonstrated there was no pleiotropic bias. Third, single SNP analysis was performed to evaluate the existence of a single SNP with abnormal impact on the results, causing result bias. Fourth, in order to assess if the connection was caused by a single SNP and to eliminate it from the follow-up study, leave-one-out sensitivity analysis and MR-PRESSO analysis were conducted.

### Multivariable mendelian randomization

We detected the overlap between SNPs of different lipid fractions (Supplementary Fig. 1). To regulate the pleiotropic mechanism that could arise from the overlap between various lipid fractions, it was necessary to estimate the impact of each lipid fraction on the outcomes, while considering the impact of other lipid fractions. We used multivariable MR analysis to overcome this problem. Specifically, we merged and reconstructed IVs using SNPs of LDL-C, HDL-C, TC, and TG, meeting our single-variable MR selected principles illustrated previously. We employed the MVMR extension of the inverse-variance weighted MR technique, along with sensitivity analyses that were robust to pleiotropy (MVMR-Egger and MVMR-Median), to evaluate the effect of each lipid fraction on the outcomes. The odds ratios (ORs) used to represent the MR estimations are used to determine how a one-unit increase in exposure (blood lipids) affects the risk of outcomes (psychiatric disorders).

### Bidirectional mendelian randomization analysis

As a supplement, we performed an additional reverse MR analysis with psychiatric illnesses as exposures and blood lipids as results to explore reverse causality. The reverse MR analysis procedure was the same as the MR analysis described earlier.

All statistical analyses were conducted employing the TwoSample MR (version 0.5.6) package and MendelianRandomization R package in R 4.1.1 (R Foundation for Statistical Computing, Vienna, Austria).

## Result

### Univariable MR analysis of blood lipids on risk of psychiatric disorders

**Fig. 2:**
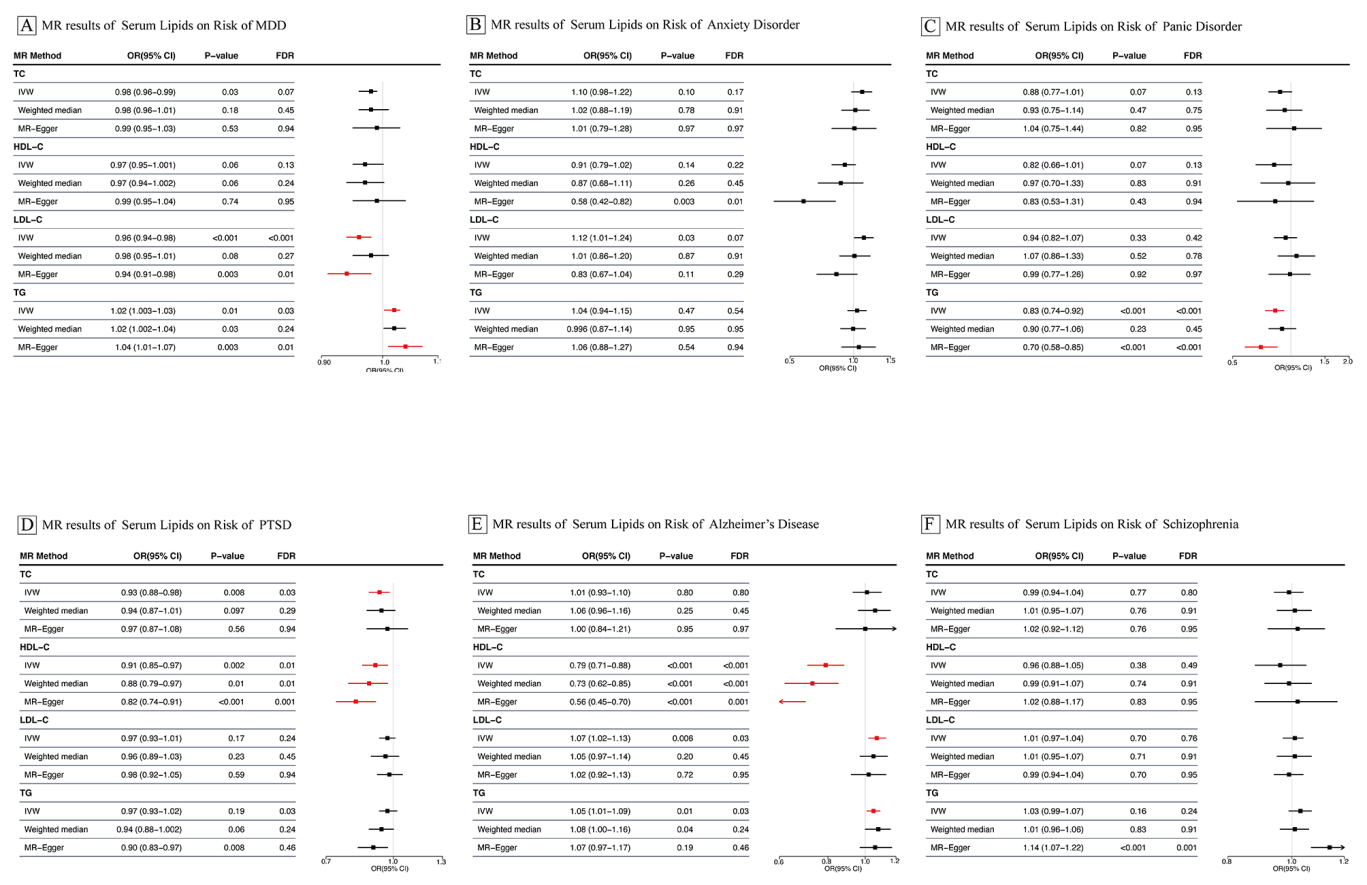
Forest Plot of Associations of blood lipids on Risk of psychiatric disorders. Abbreviations: LDL-C, Low density lipoprotein cholesterol; HDL-C, High density lipoprotein cholesterol; TC, Total cholesterol; TG, Triglycerides; MR, Mendelian randomization; IVW, inverse-variance weighted MR; CI, confidence interval; OR, odds ratio; MDD, Major Depressive Disorder; PTSD, Post-Traumatic Stress Disorder; AD, Alzheimer’s Disease. Results plotted are after pruning for instrument heterogeneity. The forest plot illustrates the OR of the impact of a 1-standard–deviation rise in genetically determined exposure (TC, HDL-C, LDL-C, TG) on outcome (psychiatric disorders) as a box, with error bars representing the 95% CI. The vertical dotted line delineates an OR of 1, i.e., no impact of the exposure on the result

**Fig. 3:**
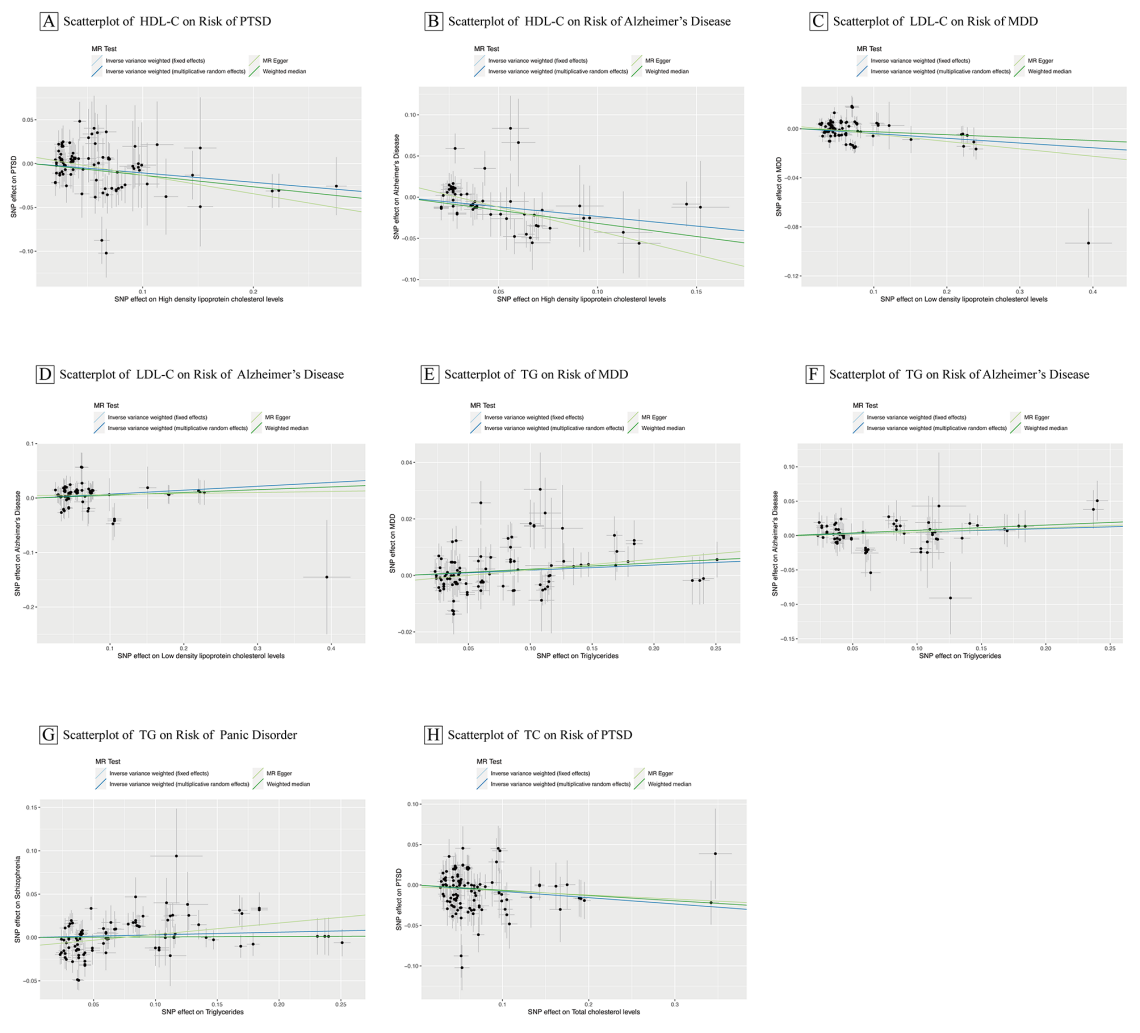
Scatter Plot of Associations of blood lipids on Risk of psychiatric disorders. Abbreviations: LDL-C, Low density lipoprotein cholesterol; HDL-C, High density lipoprotein cholesterol; TC, Total cholesterol; TG, Triglycerides; MR, Mendelian randomization; IVW, inverse-variance weighted MR; CI, confidence interval; OR, odds ratio; MDD, Major Depressive Disorder; PTSD, Post-Traumatic Stress Disorder; AD, Alzheimer’s Disease. The Scatterplot of independent instrument single-nucleotide polymorphism (SNP) exposure impacts vs. result effects from 2 independent samples augmented by the standard error of these effects on the vertical and horizontal sides (for presentation, alleles are coded so that all SNP exposure effects are positive). The solid lines denote the regression slopes fitted by the primary inverse variance–weighted (IVW) and complementary mendelian randomization (MR) approaches: slopes fitted by IVW MR method were very comparable in direction and magnitude to slopes fitted by MR-Egger and weighted median approaches for risk of the result

Results of univariable MR analysis of the associations between blood lipids and psychiatric illnesses are illustrated in Fig. 2 and Supplementary Table 2. The primary analysis results reported eight relatively robust significant associations: genetically anticipated HDL-C was causally linked to a decreased risk of AD (OR = 0.79, 95% CI = 0.71–0.88, p < 0.001) and PTSD (OR = 0.91, 95% CI = 0.85–0.97, p = 0.002); genetically predicted LDL-C was causally connected to a decreased risk of MDD (OR = 0.96, 95% CI = 0.94–0.98, p < 0.001) and an increased risk of AD (OR = 1.07, 95% CI = 1.02–1.13, p = 0.006); genetically anticipated TG was causally related to an increased risk of MDD (OR = 1.02, 95% CI = 1.003–1.03, p = 0.01), AD (OR = 1.05, 95% CI = 1.01–1.09, p = 0.01), and panic disorder (OR = 0.83, 95% CI = 0.74–0.92, p < 0.001); genetically anticipated TC was causally associated with a decreased risk of PTSD (OR = 0.93, 95% CI = 0.88–0.98, p = 0.008). A scatter plot of the eight associations mentioned earlier is shown in Fig. 3. In addition, the IVW analysis also reported two suggestive associations that were no longer significant after multiple testing correction: genetically anticipated TC was causally associated with a decreased risk of MDD (OR = 0.98, 95% CI = 0.96–0.99, p = 0.03, FDR = 0.07); genetically anticipated LDL-C was causally associated with an increased risk of anxiety disorder (OR = 1.12, 95% CI = 1.01–1.24, p = 0.03, FDR = 0.07). Notably, pleiotropy tests indicated possible bias caused by horizontal pleiotropy in the estimation of anxiety disorder, so we excluded it from our final estimates.

The results of heterogeneity and level pleiotropy tests are shown in Supplementary Table 3. For estimates with significant heterogeneity (p-value for heterogeneity < 0.05), we used the estimation outcomes of random effect model method to eliminate the bias caused by heterogeneity. The results of pleiotropy analysis showed horizontal pleiotropy in the analysis of HDL-C and AD. To eliminate possible result bias caused by horizontal pleiotropy, we adopted the estimates of MR-Egger as a reference. Although the estimation accuracy of MR-Egger is low, it provides an estimation consistent with the estimation of IVW analysis. In addition, we found and eliminated those abnormal outliers through IVW leave-one-out analysis (Supplementary File S2) and MR-PRESSO analysis (Supplementary Table 4) method to ensure that our estimates are reliable.

### Multivariable MR analysis of blood lipids Associated with Risk of psychiatric disorders

Multivariable MR analysis results of the associations between blood lipids and psychiatric disorders are shown in Table 1. The MVMR analysis results reported five significant associations, four of which were consistent with the results of univariable MR analysis: genetically anticipated HDL-C was related to the risk of AD (OR = 0.46, 95% CI = 0.32–0.65), *p* < 0.001) and PTSD (OR = 0.92, 95% CI = 0.86,0.99, *p* = 0.02) and genetically predicted TG was connected to risk of MDD (OR = 1.02, 95% CI = 1.00-1.03, *p* = 0.03) and panic disorder (OR = 0.83, 95% CI = 0.73–0.95, *p* = 0.005). In addition, the association reported at borderline significance between genetically predicted HDL-C and MDD in the univariable MR model (OR _*ivw*_=0.97, 95% CI = 0.95–1.001, *p* = 0.06) was enhanced in the MVMR model (OR = 0.98, 95% CI = 0.95–0.998, *p* = 0.03).In contrast, the connotations between genetically expected TC, LDL-C and MDD, the relations between genetically anticipated TG, LDL-C and AD, and the association among genetically expected LDL-C and AD observed in the univariable MR model were diminished and no longer significant in the MVMR model. The results obtained from methods that were robust to pleiotropy (MVMR-Egger and MVMR-Median), were generally consistent with the MVMR-IVW results.

**Table 1:** Multivariable MR Results of Blood lipids on Risk of MDD, Anxiety Disorder, Panic Disorder, PTSD, AD and SCZ

Exposure	Outcome	MVMR-IVW		MVMR-Median		MVMR-Egger	
		OR (95% CI)	P value	OR (95% CI)	P value	OR (95% CI)	P value
TC	MDD	1.02 (0.98–1.06)	0.24	1.04 (0.97–1.08)	0.28	1.01 (0.96–1.06)	0.33
	Anxiety Disorder	1.09 (0.92–1.29)	0.33	1.09 (0.90–1.30)	0.35	1.05 (0.79–1.22)	0.31
	Panic Disorder	1.14 (0.82–1.59)	0.42	1.10 (0.82–1.30)	0.40	1.04 (0.75–1.40)	0.60
	PTSD	1.02 (0.87–1.20)	0.82	1.06 (0.87–1.22)	0.88	1.01 (0.87–1.08)	0.78
	AD	1.46 (0.78–2.74)	0.24	1.30 (0.78–2.10)	0.26	1.01 (0.90–1.21)	0.30
	SCZ	0.95 (0.87–1.03)	0.23	0.99 (0.89–1.03)	0.20	1.02 (0.92–1.12)	0.26
HDL-C	MDD	**0.98 (0.95–0.998)**	**0.03**	0.96 (0.94–1.01)	0.06	**0.98 (0.96–0.99)**	**0.04**
	Anxiety Disorder	0.89 (0.75–1.05)	0.18	0.86 (0.74–1.02)	0.13	0.88 (0.76–1.05)	0.20
	Panic Disorder	0.93 (0.78–1.09)	0.36	0.90 (0.70–1.10)	0.38	0.83(0.53–1.31)	0.43
	PTSD	**0.92 (0.86–0.99)**	**0.02**	**0.90 (0.85–0.98)**	**0.02**	**0.85 (0.74–0.95)**	**0.02**
	AD	**0.46 (0.32–0.65)**	**< 0.001**	**0.56 (0.45–0.71)**	**< 0.001**	**0.58 (0.51–0.72)**	**< 0.001**
	SCZ	0.98 (0.91–1.05)	0.58	0.98 (0.91–1.07)	0.54	1.03 (0.88–1.17)	0.63
LDL-C	MDD	1.07 (0.96–1.17)	0.14	1.05 (0.96–1.17)	0.10	0.96(0.93–1.02)	0.13
	Anxiety Disorder	1.05 (0.88–1.25)	0.57	1.05 (0.90–1.22)	0.62	0.78 (0.69–1.04)	0.50
	Panic Disorder	0.99 (0.75–1.30)	0.95	1.00 (0.80–1.32)	0.98	1.00 (0.77–1.26)	0.92
	PTSD	0.95 (0.83–1.09)	0.49	0.96 (0.85–1.09)	0.55	0.96 (0.92–1.04)	0.56
	AD	0.99 (0.57–1.71)	0.97	1.00 (0.57–1.60)	0.90	1.01 (0.92–1.13)	0.89
	SCZ	1.07 (0.98–1.17)	0.14	1.05 (0.98–1.15)	0.22	0.99 (0.95–1.03)	0.10
TG	MDD	**1.02 (1.00-1.03)**	**0.03**	**1.04 (1.01–1.07)**	**0.03**	**1.05 (1.02–1.07)**	**0.026**
	Anxiety Disorder	1.03 (0.94–1.13)	0.54	1.13 (0.94–1.25)	0.61	1.05 (0.88–1.24)	0.54
	Panic Disorder	**0.83 (0.73–0.95)**	**0.005**	**0.86 (0.73–0.98)**	**0.004**	**0.78(0.62–0.85)**	**0.004**
	PTSD	0.98 (0.93–1.03)	0.45	0.95 (0.93–1.01)	0.56	0.87 (0.80–0.97)	0.52
	AD	0.93 (0.72–1.20)	0.57	0.90 (0.72–1.16)	0.55	1.07 (0.97–1.17)	0.50
	SCZ	1.05 (0.999-1.10)	0.05	1.02 (0.98–1.10)	0.06	1.04 (0.96–1.22)	0.06

Abbreviations: TC, Total cholesterol; HDL-C, High density lipoprotein cholesterol; LDL-C, Low density lipoprotein cholesterol; TG, Triglycerides; MDD, Major Depressive Disorder; PTSD, Post-Traumatic Stress Disorder; AD, Alzheimer’s Disease; SCZ, Schizophrenia; OR, odds ratio

Each estimate (OR) is based on the multivariable IVW method and represents the direct effect of the risk factor on the respective outcome after controlling for the other 3 lipid fraction in MVMR. ORs are reported per SD increase in the respective lipid fraction

Associations of P < 0.05 are shown in bold

### MR analysis of psychiatric disorders on risk of blood lipids

MR analysis outcomes of the causal relationship between psychiatric disorders and blood lipids are shown in Supplementary Table 5. As there were inadequate genome-wide significant SNPs for Anxiety Disorder, Panic Disorder and PTSD that could be used as instrumental variables, we only evaluated the causal impacts of MDD, AD and Schizophrenia on blood lipids. The results reported a significant relationship among genetically anticipated MDD and TG risk (OR = 1.10, 95% CI = 1.02–1.18, *p* = 0.01). The estimate was mostly in line with results from the MR-Egger sensitivity and weighted median analyses.

## Discussion

In this investigation, we assessed potential bidirectional relations among the major fractions of blood lipids and six psychiatric disorders (MDD, anxiety disorder, panic disorder, PTSD, AD and schizophrenia). We used the latest GWAS statistics of blood lipids, with a large number of samples, as the data source of exposure. We also used GWAS statistics of psychiatric disorders from PGC and ADGC, which are regarded as authoritative institutions for the study of psychiatric disorders. We performed univariable MR analysis, multivariable MR analysis and rigorous multiple sensitivity analysis to ensure the reliability of the results. To the best of our knowledge, this is the first MR analysis to find genetic confirmation of the causal impacts of blood lipids on the risk of Anxiety Disorder, PTSD and Panic Disorder. Associations between blood lipids and MDD, AD and Schizophrenia have been informed in previous MR investigations [15–19]. Nevertheless, most of these studies are univariable analyses, the datasets are outdated, and the conclusions are incomplete or even conflicting. Thus, further and more comprehensive research is very necessary.

The associations between blood lipids and psychiatric disorders have been widely studied, including the mechanism between them. Cholesterol is extensively distributed in all tissues, especially in the nervous system. It has a vital function in the growth, function, and stability of synapses [33]. Studies have shown that reduced brain cholesterol levels and synapses may be the characteristics of mood disorders [33]. The level of TG is negatively correlated with the thickness of the anterior cingulate cortex [34], which participates in emotional assessment, emotion-related learning and autonomic regulation [35]. Recent investigations have revealed that lipid metabolism is closely connected to monoamine neurotransmitters, and monoaminergic neurotransmission dysfunction is closely associated with psychiatric disorders [36]. This explains some possible mechanisms between dyslipidemia and mental diseases. However, it is not clear which changes in blood lipids or psychiatric disorders occur first, and the causal relationship remains to be clarified. Our work builds on the proof of principle and provides supplementary evidence of the mechanism that may link blood lipids with various psychiatric disorders.

We found a causal connection between TG level and MDD in this investigation. The finding largely agrees with those reported in earlier researches. For Instance, in a 2020 meta-analysis of patients with first-onset depression, the authors found that increased TG level was associated with first-time depression [37]. A recently published study [38] reported that higher TG level mediated the relationship between lack of recreational activities and sedentary play and high risk of depression. The association between depression and blood cholesterol levels is complex, and, so far, there is no definite conclusion. Our univariable MR analysis revealed that higher LDL-C levels were associated with a lower risk of MDD, while no significant associations were found for HDL-C and TC levels. However, after adjusting with multivariable MR model, we found that HDL-C levels were associated with a lower risk of MDD. Our outcomes are not fully agreed with the results of previous studies [16, 18, 39–41]. This discordance may be attributed to differences in research methods, sample size, or the definition of demarcation points for lipoprotein-C. Notably, compared with previous MR studies, we adopted a multivariable approach to avoid possible pleiotropic effects between different blood lipid fractions and improve the accuracy of estimation.

Previous observational investigations have informed a relationship between blood lipids and anxiety disorder. Nevertheless, we did not discover a significant causal connection between blood lipids and anxiety disorder. A 2012 study [9] reported that higher HDL-C and lower LDL-C have a positive regulatory effect on depression and anxiety. In a 2021 study [5], the authors found that intervention in triglyceride level can effectively alleviate anxiety symptoms. A recently published animal experimental study also reported that eight weeks of an increased-fat diet effectively caused metabolic disorders, like obesity and hyperlipidemia, resulting in anxiety-like behavior in mice [42]. In the recent investigation, we employed MR analysis for the first time to assess the relationship between blood lipids and anxiety disorder and discovered no genetic evidence for the causal association between them. This shows that the associations found in previous observational studies do not show causality. Remarkably, we found a small number of SNP outliers, which subversively change the overall results between TG and anxiety disorder. This finding further suggests that the associations found in previous observational studies may be caused by common pleiotropic SNPs. The potential impact and possible mechanism of these gene loci deserve further studies.

Research on the relationship between blood lipids and panic disorder is insufficient. To the best of our knowledge, no previous MR study has inspected the connection between blood lipids and panic disorder. We discovered a novel substantial negative relation between TG level and panic disorder risk. This suggests that higher TG level has a protective effect on panic disorder. The estimate was consistent across univariable and multivariable and MR-Egger sensitivity analyses. In Addition, a previous observational study informed a positive correlation between cholesterol levels and panic disorder in women [43]. However, such an association was not found in our study, suggesting that the possible association between cholesterol and panic disorder may not be a causal one.

For the first time, we used MR analysis method to find possible genetic evidence for the relationship between blood lipids and PTSD. Our study detected a significant relationship between HDL-C level and PTSD risk. The increase in HDL-C level can decrease PTSD risk. Because of the limitations of observational studies, conclusions on the relationship between blood lipids and PTSD are inconsistent [6, 10, 44]. In a 2014 study, the authors found that the higher the TC, the lower the suicidal ideation, aggression and associated depressive symptoms in patients with PTSD [44]. Some studies have shown that changes in lipid components in the brain may have a great impact on the integrity of cell membrane structure and function, eventually leading to abnormal neurotransmitter signals [45, 46]. This change in neurotransmitter signaling may, in turn, promote depression, PTSD and suicidal behavior. Similar to the results of earlier studies, we found that a rise in TC level suppressed PTSD risk in univariate MR analysis, but this relation was not significant after the adjustment of multivariable MR model. However, the relation between HDL-C level and PTSD risk was significant in both univariable and multivariable MR analysis. This suggests that the impact of TC on PTSD risk may be attributed to the common pleiotropic SNPs of TC and HDL-C.

In the investigation of the association between blood lipids and AD, we found that HDL-C and TG were significantly connected to AD risk. The connection between cholesterol and AD has been reported many times in previous researches. A 2019 case-control study found that higher LDL-C l and TC levels and lesser HDL-C levels were connected to AD risk [47]. A 2020 study found that PUTG component scores were significantly associated with AD biomarkers [48]. A recent MR study in 2021 found that higher HDL-C levels can reduce the risk of AD [15]. Our findings are basically consistent with previous conclusions, but the connection between LDL-C and TC and AD risk found in a few studies has not been replicated in our study. This relationship may not be a causal one. Studies on the relationship between TG and AD have also been reported in recent years. In a study of risk factors for cardiovascular disease and AD, the authors found a possible association between TG and AD [7]. A recent study showed that careful management of cholesterol and TG levels in early adulthood can reduce AD risk [49]. Our study also found a significant relationship between TG and AD. However, it should be noted that the significant association between TG and Alzheimer’s disease only exists in univariate MR analysis. After the adjustment of the multivariate MR model, no significant association was found between TG and AD. This suggests that the association between TG and AD observed in prior investigations may be affected by the pleiotropic pathways of various components of blood lipids.

We found no significant association between blood lipids and schizophrenia, which is consistent with a recent clinical controlled trial [11]. In our study, univariable MR-Egger analysis indicated a significant association between TG and schizophrenia. After adjusting the multivariate MR model, the association was found to be at borderline significance (OR = 1.05; 95% CI = 0.9996-1.10; *p* = 0.05). Though the relationship cannot be included in the final result, as a reference, we must consider that there may be potential associations yet to be revealed. More in-depth research on the relationship between blood lipids and schizophrenia are needed in the future.

### Strengths and limitations

The research we conducted has a number of advantages. First, we employed the most recent summaries of genetic associations from the largest GWASs that were available. Larger sample sizes often improve measurement accuracy. Second, one of the primary strengths of our MVMR models was that they allowed us to take into consideration possible pleiotropic mechanisms that might result from the overlap between various lipid fractions. Third, multiple steps were undertaken to eliminate the bias caused by heterogeneity and horizontal pleiotropy. Fourth, the results were broadly consistent across sensitivity analyses. However, this study also has some restrictions. First, our information was from European populations, which may limit the application of the conclusions to other populations, such as Asians and Africans. Second, although we used sensitivity analyses to minimize the probability of violating MR assumptions, we cannot completely rule out the possibility of residual pleiotropy. Therefore, potential pleiotropic SNPs, one of the most common challenges in MR research, may affect our results.

## Conclusions

In summary, we evaluated the two-way causal relationships between blood lipids and psychiatric disorders. We found specific effects of blood lipids levels on MDD, PTSD, AD and panic disorder using MR analysis, with some of these associations reported here for the first time. We found that genetically predicted HDL-C level is a protective factor for AD and PTSD; genetically predicted TG level is a protective factor for panic disorder; and genetically predicted TG level is a risk factor for MDD. Additionally, we also found that the occurrence of MDD can lead to higher TG level using reverse analysis. These findings provide an essential reference value for early risk assessment, intervention and treatment of dyslipidemia and psychiatric disorders. Future research should explore the mechanism underlying this causality to develop prevention and treatment strategies that could potentially change the interaction between blood lipid levels and psychiatric disorders.

### Electronic supplementary material

Below is the link to the electronic supplementary material.

Supplementary Material 1

Supplementary Material 2

Supplementary Material 3

Supplementary Material 4

Supplementary Material 5

Supplementary Material 6

## Data Availability

All data used for the analyses are publicly available. All data of psychiatric disorders can be obtained from Psychiatric Genomics Consortium (https://www.med.unc.edu/pgc/download-results/). Blood lipids data can be downloaded from GWAS Catalog (https://www.ebi.ac.uk/gwas/home) according to PubMed ID = 29,507,422.
