# Supplementary material for: Causal relationships between blood lipids and major psychiatric disorders: Univariable and multivariable mendelian randomization analysis
Source: BMC Med Genomics. 2023 Oct 18;16:250. doi: 10.1186/s12920-023-01692-8 (PMC10585856; doi:10.1186/s12920-023-01692-8)
Supplement: Supplementary file 4 — Supplementary Material 4 [file 12920_2023_1692_MOESM4_ESM.docx]

**STROBE-MR checklist**

| **Item No.** | **Section** | **Checklist item** | **Page No.** | **Relevant text from manuscript** |
| --- | --- | --- | --- | --- |
| 1 | **TITLE and ABSTRACT** | Indicate Mendelian randomization (MR) as the study’s design in the title and/or the abstract if that is a main purpose of the study | 1 | Complete |
|  | **INTRODUCTION** |  |  |  |
| 2 | **Background** | Explain the scientific background and rationale for the reported study. What is the exposure? Is a potential causal relationship between exposure and outcome plausible? Justify why MR is a helpful method to address the study question | 2-3 | The scientific background and rationale for our research is described in the Introduction in the first two paragraphs. We then move on and describe the importance of MR in paragraph three. |
| 3 | **Objectives** | State specific objectives clearly, including pre-specified causal hypotheses (if any). State that MR is a method that, under specific assumptions, intends to estimate causal effects | 5 | Objectives are clearly described at the end of the 4^rd^ paragraph in the Introduction. |
|  | **METHODS** |  |  |  |
| 4 | **Study design and data sources** | Present key elements of the study design early in the article. Consider including a table listing sources of data for all phases of the study. For each data source contributing to the analysis, describe the following: | 6 | Supplementary Table 1 |
|  | a) | Setting: Describe the study design and the underlying population, if possible. Describe the setting, locations, and relevant dates, including periods of recruitment, exposure, follow-up, and data collection, when available. | 6-7 | Described in the “Study design” and “Source of outcomes” section |
|  | b) | Participants: Give the eligibility criteria, and the sources and methods of selection of participants. Report the sample size, and whether any power or sample size calculations were carried out prior to the main analysis | 6-7 | Resource on Adult Health and Aging (GERA) cohort (n = 94,674 patients with untreated lipid values) provided genome-wide association data for HDL, LDL, TG, and TC(21). Over 20 million SNPs and 478,866 longitudinal….”  Utilizing genetic data from the European population based on exposure data helped to eliminate possible bias caused by population heterogeneity. The following were the respective sample sizes |
|  | c) | Describe measurement, quality control and selection of genetic variants | 6 | Described in the “Instrumental variable” selection |
|  | d) | For each exposure, outcome, and other relevant variables, describe methods of assessment and diagnostic criteria for diseases | 7 |  |
|  | e) | Provide details of ethics committee approval and participant informed consent, if relevant | 6 | “No institutional review board authorization was needed for this study's ethical….” |
| 5 | **Assumptions** | Explicitly state the three core IV assumptions for the main analysis (relevance, independence and exclusion restriction) as well assumptions for any additional or sensitivity analysis | 5 | Described in the "Study design” section in the Methods. |
| 6 | **Statistical methods: main analysis** | Describe statistical methods and statistics used | 7-9 | All main statistical methods are reported in the methods under the section ‘Univariable MR analysis and Multivariable MR analysis’. These include how genetic variants were selected, the statistical methods that were used for each model and the covariates used. |
|  | a) | Describe how quantitative variables were handled in the analyses (i.e., scale, units, model) | 7 |  |
|  | b) | Describe how genetic variants were handled in the analyses and, if applicable, how their weights were selected | 8 |  |
|  | c) | Describe the MR estimator (e.g. two-stage least squares, Wald ratio) and related statistics. Detail the included covariates and, in case of two-sample MR, whether the same covariate set was used for adjustment in the two samples | 7 |  |
|  | d) | Explain how missing data were addressed |  |  |
|  | e) | If applicable, indicate how multiple testing was addressed | 9 | For multiple comparisons, the Benjamini & Hochberg….. |
| 7 | **Assessment of assumptions** | Describe any methods or prior knowledge used to assess the assumptions or justify their validity | 10 | “We performed various tests to evaluate potential IV violations…..” |
| 8 | **Sensitivity analyses and additional analyses** | Describe any sensitivity analyses or additional analyses performed (e.g. comparison of effect estimates from different approaches, independent replication, bias analytic techniques, validation of instruments, simulations) | 9-10 | Weighted Median  MR-Egger  Multivariable MR  Bidirectional MR  MR-PRESSO |
| 9 | **Software and pre-registration** |  |  |  |
|  | a) | Name statistical software and package(s), including version and settings used | 11 | “All statistical analyses were conducted employing the TwoSample MR…..” |
|  | b) | State whether the study protocol and details were pre-registered (as well as when and where) | - | NA |
|  | **RESULTS** |  |  |  |
| 10 | **Descriptive data** |  |  |  |
|  | a) | Report the numbers of individuals at each stage of included studies and reasons for exclusion. Consider use of a flow diagram | - | Fig 1 |
|  | b) | Report summary statistics for phenotypic exposure(s), outcome(s), and other relevant variables (e.g. means, SDs, proportions) | 6 | NA |
|  | c) | If the data sources include meta-analyses of previous studies, provide the assessments of heterogeneity across these studies |  | NA |
|  | d) | For two-sample MR:  i.  Provide justification of the similarity of the genetic variant-exposure associations between the exposure and outcome samples  ii.  Provide information on the number of individuals who overlap between the exposure and outcome studies | 7 | Sample Independence |
| 11 | **Main results** |  |  |  |
|  | a) | Report the associations between genetic variant and exposure, and between genetic variant and outcome, preferably on an interpretable scale | 11 |  |
|  | b) | Report MR estimates of the relationship between exposure and outcome, and the measures of uncertainty from the MR analysis, on an interpretable scale, such as odds ratio or relative risk per SD difference | 11 | Results of univariable MR analysis of the associations between blood lipids….. |
|  | c) | If relevant, consider translating estimates of relative risk into absolute risk for a meaningful time period |  | NA |
|  | d) | Consider plots to visualize results (e.g. forest plot, scatterplot of associations between genetic variants and outcome versus between genetic variants and exposure) | 11-12 | We visualize results from the main univariable using a forest plot in Fig2 and a scatter plot in Fig3 |
| 12 | **Assessment of assumptions** |  |  |  |
|  | a) | Report the assessment of the validity of the assumptions | 11 | Described in ‘The results of heterogeneity and level pleiotropy tests’ section |
|  | b) | Report any additional statistics (e.g., assessments of heterogeneity across genetic variants, such as *I^2^*, Q statistic or E-value) | 11 | The results of heterogeneity and level pleiotropy tests are shown in Supplementary Table 3. |
| 13 | **Sensitivity analyses and additional analyses** |  |  |  |
|  | a) | Report any sensitivity analyses to assess the robustness of the main results to violations of the assumptions | 11-12 | Weighted Median, MR-Egger 、MR-PRESSO and Multivariable MR were performed to assess the robustness of the main results. |
|  | b) | Report results from other sensitivity analyses or additional analyses |  |  |
|  | c) | Report any assessment of direction of causal relationship (e.g., bidirectional MR) | 13 | Described in ‘MR Analysis of Psychiatric Disorders on Risk of Blood Lipids’ section |
|  | d) | When relevant, report and compare with estimates from non-MR analyses |  | NA |
|  | e) | Consider additional plots to visualize results (e.g., leave-one-out analyses) | 12 | Supplement file S3 S4 |
|  | **DISCUSSION** |  |  |  |
| 14 | **Key results** | Summarize key results with reference to study objectives | 14 | We describe key results in the first paragraph of the discussion section. |
| 15 | **Limitations** | Discuss limitations of the study, taking into account the validity of the IV assumptions, other sources of potential bias, and imprecision. Discuss both direction and magnitude of any potential bias and any efforts to address them | 19 | Strengths and Limitations |
| 16 | **Interpretation** |  |  |  |
|  | a) | Meaning: Give a cautious overall interpretation of results in the context of their limitations and in comparison with other studies | 14-18 | Discussion – paragraph 3-8 |
|  | b) | Mechanism: Discuss underlying biological mechanisms that could drive a potential causal relationship between the investigated exposure and the outcome, and whether the gene-environment equivalence assumption is reasonable. Use causal language carefully, clarifying that IV estimates may provide causal effects only under certain assumptions | 15 | Discussion – paragraph 2 |
|  | c) | Clinical relevance: Discuss whether the results have clinical or public policy relevance, and to what extent they inform effect sizes of possible interventions | 20 | Conclusions |
| 17 | **Generalizability** | Discuss the generalizability of the study results (a) to other populations, (b) across other exposure periods/timings, and (c) across other levels of exposure |  | NA |
|  | **OTHER INFORMATION** |  |  |  |
| 18 | **Funding** | Describe sources of funding and the role of funders in the present study and, if applicable, sources of funding for the databases and original study or studies on which the present study is based | 21 | Financial support |
| 19 | **Data and data sharing** | Provide the data used to perform all analyses or report where and how the data can be accessed, and reference these sources in the article. Provide the statistical code needed to reproduce the results in the article, or report whether the code is publicly accessible and if so, where | 21 | Associated Data |
| 20 | **Conflicts of Interest** | All authors should declare all potential conflicts of interest | 21 | Conflict of interest |

This checklist is copyrighted by the Equator Network under the Creative Commons Attribution 3.0 Unported (CC BY 3.0) license.
