# Supplementary figures and images for: Causal relationships between blood lipids and major psychiatric disorders: Univariable and multivariable mendelian randomization analysis
Source: BMC Med Genomics. 2023 Oct 18;16:250. doi: 10.1186/s12920-023-01692-8 (PMC10585856; doi:10.1186/s12920-023-01692-8)

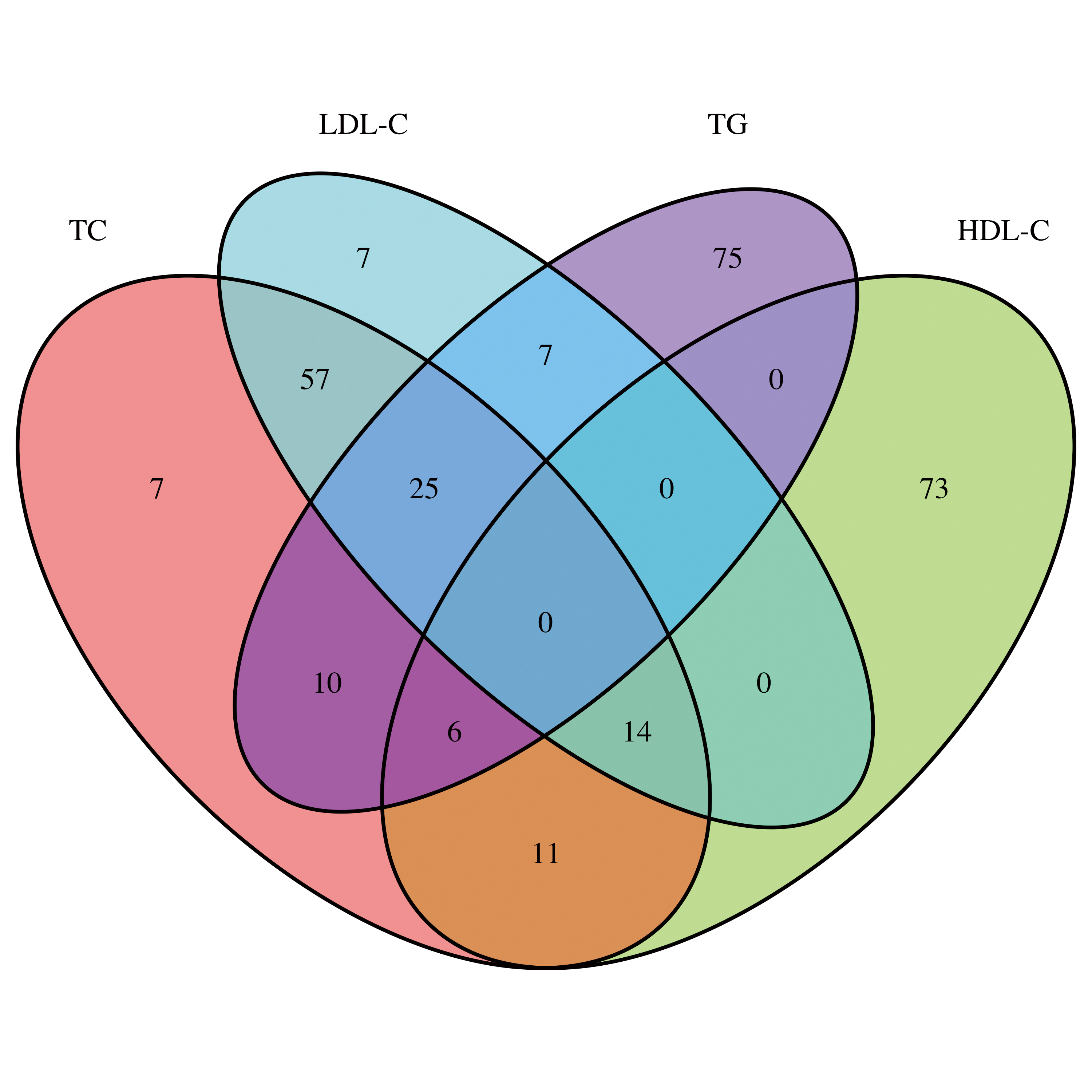

Supplement: Supplementary file 5 — Supplementary Material 5 [file 12920_2023_1692_MOESM5_ESM.jpg]
