# Supplementary material for: Causal relationships between blood lipids and major psychiatric disorders: Univariable and multivariable mendelian randomization analysis
Source: BMC Med Genomics. 2023 Oct 18;16:250. doi: 10.1186/s12920-023-01692-8 (PMC10585856; doi:10.1186/s12920-023-01692-8)
Supplement: Supplementary file 6 — Supplementary Material 6 [file 12920_2023_1692_MOESM6_ESM.docx]

**Supplementary Materials**

**Supplementary Table 1.** Characteristics of included genome-wide association studies for serum lipids and mental disorders.

**Supplementary Table 2.** Univariable MR Results of Blood lipids on Risk of MDD and Anxiety Disorder and Panic Disorder and PTSD and AD and SCZ.

**Supplementary Table 3.** Results of potential pleiotropy and heterogeneity assessments.

**Supplementary Table 4**. MR-PRESSO analysis results of Blood lipids on Risk of MDD and Anxiety Disorder and Panic Disorder and PTSD and AD and SCZ

**Supplementary Table 5**. MR Results of MDD/AD/SCZ on Risk of Blood lipids

**Supplementary Fig 1**. Venn plot of instrument variables of 4 fractions of blood lipids

| Exposure/outcome | GWAS Catalog  accession number | PMID | Participants | Population | Web source |
| --- | --- | --- | --- | --- | --- |
| **Exposure** |  |  |  |  |  |
| Total cholesterol | GCST007143 | 29507422 | 76,627 individuals * | European | https://www.ebi.ac.uk/gwas/studies/GCST007143 |
| HDL cholesterol | GCST007140 | 29507422 | 76,627 individuals * | European | https://www.ebi.ac.uk/gwas/studies/GCST007140 |
| LDL cholesterol | GCST007141 | 29507422 | 76,627 individuals * | European | https://www.ebi.ac.uk/gwas/studies/GCST007141 |
| Triglycerides | GCST007142 | 29507422 | 76,627 individuals * | European | https://www.ebi.ac.uk/gwas/studies/GCST007142 |
| **Outcome** |  |  |  |  |  |
| Major depressive disorder | GCST007342 | 30718901 | 170,756 cases, 329,443 controls****** | European | <https://www.med.unc.edu/pgc/download-results/> |
| Anxiety disorder | GCST003370 | 26754954 | 7016 cases, 14,745 controls | European | <https://www.med.unc.edu/pgc/download-results/> |
| Panic disorder | GCST009525 | 31712720 | 2408 cases, 228,470 controls | European | <https://www.med.unc.edu/pgc/download-results/> |
| Posttraumatic stress disorder | GCST009315 | 31594949 | 32,428 cases,174,227 controls | European | <https://www.med.unc.edu/pgc/download-results/> |
| Alzheimer's Disease | GCST007511 | 30820047 | 21,982 cases, 41,944 controls | European | https://gwas.mrcieu.ac.uk/datasets/ieu-b-2/ |
| Schizophrenia | GCST002539 | 25056061 | 36,989 cases, 113,075 controls | European | <https://www.med.unc.edu/pgc/download-results/> |

**Supplementary Table 1.** Characteristics of included genome-wide association studies for serum lipids and mental disorders

Abbreviations：TC, Total cholesterol; HDL-C, High density lipoprotein cholesterol; LDL-C, Low density lipoprotein cholesterol; TG, Triglycerides.

* The blood lipids sample included participants of European ancestry only.

** The sample size of major depression is the sample size after excluding the data of 23andMe.

**Supplementary Table 2.** Univariable MR Results of Blood lipids on Risk of MDD and Anxiety Disorder and Panic Disorder and PTSD and AD and SCZ

| Exposure | Outcome |  |  | IVW | | |  |  |  | Weighted median | | |  |  | MR Egger | | |  |
| --- | --- | --- | --- | --- | --- | --- | --- | --- | --- | --- | --- | --- | --- | --- | --- | --- | --- | --- |
|  |  | OR (95% CI) | | | P value | FDR^#^ | | OR (95% CI) | | | P value | FDR^#^ | | OR (95% CI) | | P value | FDR^#^ | |
| TC | MDD | **0.98 (0.96-0.99) ^b^** | | | **0.03** | 0.07 | | 0.98 (0.96-1.01) | | | 0.18 | 0.45 | | 0.99 (0.95-1.03) | | 0.53 | 0.94 | |
|  | Anxiety Disorder | 1.10 (0.98-1.22) ^b^ | | | 0.10 | 0.17 | | 1.02 (0.88-1.19) | | | 0.78 | 0.91 | | 1.01 (0.79-1.28) | | 0.97 | 0.97 | |
|  | Panic Disorder | 0.88 (0.77-1.01) ^a^ | | | 0.07 | 0.13 | | 0.93(0.75-1.14) | | | 0.47 | 0.75 | | 1.04(0.75-1.44) | | 0.82 | 0.95 | |
|  | PTSD | **0.93 (0.88-0.98) ^b^** | | | **0.008** | **0.03** | | 0.94 (0.87-1.01) | | | 0.097 | 0.29 | | 0.97 (0.87-1.08) | | 0.56 | 0.94 | |
|  | AD | 1.01 (0.93-1.10) ^b^ | | | 0.80 | 0.80 | | 1.06 (0.96-1.16) | | | 0.25 | 0.45 | | 1.00 (0.84-1.21) | | 0.96 | 0.97 | |
|  | SCZ | 0.99 (0.94-1.04) ^b^ | | | 0.77 | 0.80 | | 1.01 (0.95-1.07) | | | 0.76 | 0.91 | | 1.02 (0.92-1.12) | | 0.76 | 0.95 | |
| HDL-C | MDD | 0.97 (0.95-1.001) ^b^ | | | 0.06 | 0.13 | | 0.97 (0.94-1.002) | | | 0.06 | 0.24 | | 0.99 (0.95-1.04) | | 0.74 | 0.95 | |
|  | Anxiety Disorder | 0.91 (0.79-1.02) ^a^ | | | 0.14 | 0.22 | | 0.87(0.68-1.11) | | | 0.26 | 0.45 | | **0.58 (0.42-0.82)** | | **0.003** | **0.01** | |
|  | Panic Disorder | 0.82 (0.66-1.01) ^a^ | | | 0.07 | 0.13 | | 0.97(0.70-1.33) | | | 0.83 | 0.91 | | 0.83(0.53-1.31) | | 0.43 | 0.94 | |
|  | PTSD | **0.91 (0.85-0.97) ^a^** | | | **0.002** | **0.01** | | **0.88 (0.79-0.97)** | | | **0.01** | 0.12 | | **0.82 (0.74-0.91)** | | **<0.001** | **0.001** | |
|  | AD | **0.79 (0.71-0.88) ^a^** | | | **<0.001** | **<0.001** | | **0.73 (0.62-0.85)** | | | **<0.001** | 0.002 | | **0.56 (0.45-0.70)** | | **<0.001** | **0.001** | |
|  | SCZ | 0.96 (0.88-1.05) ^b^ | | | 0.38 | 0.46 | | 0.99 (0.91-1.07) | | | 0.74 | 0.91 | | 1.02 (0.88-1.17) | | 0.83 | 0.95 | |
| LDL-C | MDD | **0.96 (0.94-0.98) ^b^** | | | **<0.001** | **<0.001** | | 0.98 (0.95-1.01) | | | 0.08 | 0.27 | | **0.94 (0.91-0.98)** | | **0.003** | **0.01** | |
|  | Anxiety Disorder | **1.12 (1.01-1.24) ^a^** | | | **0.03** | 0.07 | | 1.01 (0.86-1.20) | | | 0.87 | 0.91 | | 0.83 (0.67-1.04) | | 0.11 | 0.29 | |
|  | Panic Disorder | 0.94 (0.82-1.07) ^a^ | | | 0.33 | 0.42 | | 1.07(0.86-1.33) | | | 0.52 | 0.78 | | 0.99(0.77-1.26) | | 0.92 | 0.97 | |
|  | PTSD | 0.97 (0.93-1.01) ^a^ | | | 0.17 | 0.24 | | 0.96 (0.89-1.03) | | | 0.23 | 0.45 | | 0.98 (0.92-1.05) | | 0.59 | 0.94 | |
|  | AD | **1.07 (1.02-1.13) ^a^** | | | **0.006** | **0.03** | | 1.05 (0.97-1.14) | | | 0.20 | 0.45 | | 1.02 (0.92-1.13) | | 0.72 | 0.95 | |
|  | SCZ | 1.01 (0.97-1.04) ^a^ | | | 0.70 | 0.76 | | 1.01 (0.95-1.07) | | | 0.71 | 0.91 | | 0.99 (0.94-1.04) | | 0.70 | 0.95 | |
| TG | MDD | **1.02 (1.003-1.03) ^b^** | | | **0.01** | **0.03** | | **1.02 (1.002-1.04)** | | | **0.03** | 0.24 | | **1.04 (1.01-1.07)** | | **0.003** | **0.01** | |
|  | Anxiety Disorder | 1.04 (0.94-1.15) ^a^ | | | 0.47 | 0.54 | | 0.996 (0.87-1.14) | | | 0.95 | 0.95 | | 1.06 (0.88-1.27) | | 0.54 | 0.94 | |
|  | Panic Disorder | **0.83 (0.74-0.92) ^a^** | | | **<0.001** | **<0.001** | | 0.90(0.77-1.06) | | | 0.23 | 0.45 | | **0.70(0.58-0.85)** | | **<0.001** | **0.001** | |
|  | PTSD | 0.97 (0.93-1.02) ^a^ | | | 0.19 | 0.25 | | 0.94 (0.88-1.002) | | | 0.06 | 0.24 | | **0.90 (0.83-0.97)** | | **0.008** | **0.02** | |
|  | AD | **1.05 (1.01-1.09) ^a^** | | | **0.01** | **0.03** | | **1.08 (1.00-1.16)** | | | **0.04** | 0.24 | | 1.06 (0.97-1.17) | | 0.19 | 0.46 | |
|  | SCZ | 1.03 (0.99-1.07) ^b^ | | | 0.16 | 0.24 | | 1.01 (0.96-1.06) | | | 0.83 | 0.91 | | **1.14 (1.07-1.22)** | | **<0.001** | **0.001** | |

Abbreviations: TC, Total cholesterol; HDL-C, High density lipoprotein cholesterol; LDL-C, Low density lipoprotein cholesterol; TG, Triglycerides; MDD, Major Depressive Disorder; PTSD, Post-Traumatic Stress Disorder; AD, Alzheimer's Disease; SCZ, Schizophrenia; OR, odds ratio; FDR, false discovery rate.

Associations of P < 0.05 are shown in bold.

^#^ Benjamini-Hochberg multiple testing correction.

^a^ estimates of fixed-effect model; ^b^ estimates of random-effect model.

**Supplementary Table 3.** Results of potential pleiotropy and heterogeneity assessments

| Exposure | Outcome | Q value for heterogeneity | P-value for heterogeneity | P-value for intercept |
| --- | --- | --- | --- | --- |
| TC | MDD | 209 | **<0.001** | 0.62 |
|  | Anxiety Disorder | 134 | **0.047** | 0.42 |
|  | Panic Disorder | 113 | 0.35 | 0.28 |
|  | PTSD | 143 | **0.04** | 0.37 |
|  | AD | 159 | **<0.001** | 0.32 |
|  | SCZ | 211 | **<0.001** | 0.60 |
| HDL-C | MDD | 173 | **<0.001** | 0.27 |
|  | Anxiety Disorder | 49 | 0.997 | **0.004** |
|  | Panic Disorder | 73 | 0.87 | 0.93 |
|  | PTSD | 97 | 0.44 | **0.02** |
|  | AD | 67 | 0.26 | **<0.001** |
|  | SCZ | 296 | **<0.001** | 0.34 |
| LDL-C | MDD | 151 | **<0.001** | 0.22 |
|  | Anxiety Disorder | 78 | 0.81 | **0.004** |
|  | Panic Disorder | 92 | 0.51 | 0.62 |
|  | PTSD | 100 | 0.45 | 0.67 |
|  | AD | 51 | 0.91 | 0.71 |
|  | SCZ | 87 | 0.75 | 0.43 |
| TG | MDD | 131 | **0.02** | 0.06 |
|  | Anxiety Disorder | 123 | 0.04 | 0.80 |
|  | Panic Disorder | 69 | 0.997 | 0.051 |
|  | PTSD | 128 | 0.09 | **0.02** |
|  | AD | 42 | 0.99 | 0.95 |
|  | SCZ | 168 | **<0.001** | **<0.001** |

Abbreviations: TC, Total cholesterol; HDL-C, High density lipoprotein cholesterol; LDL-C, Low density lipoprotein cholesterol; TG, Triglycerides; MDD, Major Depressive Disorder; PTSD, Post-Traumatic Stress Disorder; AD, Alzheimer's Disease; SCZ, Schizophrenia; OR, odds ratio.

P-value for heterogeneity < 0.05 indicates the presence of heterogeneity, shown in bold.

P-value for intercept < 0.05 indicates the presence of pleiotropy, shown in bold.

**Supplementary Table 4.** MR-PRESSO analysis results of Blood lipids on Risk of MDD and Anxiety Disorder and Panic Disorder and PTSD and AD and SCZ

| Exposure | Outcome | MR Analysis | Causal Estimate | SD | T-stat | P-value | Remove SNP |
| --- | --- | --- | --- | --- | --- | --- | --- |
| TC | MDD | MR-PRESSO | -0.016 | 0.01 | -1.58 | 0.12 | 2 SNP |
|  | MDD | Outlier-corrected MR-PRESSO | -0.012 | 0.009 | -1.24 | 0.22 | - |
|  | Anxiety Disorder | MR-PRESSO | 0.12 | 0.05 | 2.21 | 0.03 | - |
|  | Panic Disorder | MR-PRESSO | -0.11 | 0.07 | -1.55 | 0.12 | - |
|  | PTSD | MR-PRESSO | -0.07 | 0.03 | -2.64 | 0.009 | - |
|  | AD | MR-PRESSO | 0.01 | 0.04 | 0.33 | 0.74 | 3 SNP |
|  | AD | Outlier-corrected MR-PRESSO | 0.02 | 0.03 | 0.67 | 0.50 | - |
|  | SCZ | MR-PRESSO | 0.004 | 0.25 | 0.17 | 0.87 | 2 SNP |
|  | SCZ | Outlier-corrected MR-PRESSO | 0.016 | 0.02 | 0.84 | 0.40 | - |
| HDL-C | MDD | MR-PRESSO | -0.03 | 0.01 | -1.92 | 0.056 | 2 SNP |
|  | MDD | Outlier-corrected MR-PRESSO | -0.02 | 0.01 | -1.73 | 0.09 | - |
|  | Anxiety Disorder | MR-PRESSO | -0.10 | 0.07 | -1.49 | 0.14 | - |
|  | Panic Disorder | MR-PRESSO | -0.19 | 0.10 | -1.91 | 0.058 | - |
|  | PTSD | MR-PRESSO | -0.11 | 0.03 | -3.25 | 0.002 | - |
|  | AD | MR-PRESSO | -0.10 | 0.04 | -2.48 | 0.01 | 2 SNP |
|  | AD | Outlier-corrected MR-PRESSO | -0.12 | 0.04 | -3.30 | 0.001 | - |
|  | SCZ | MR-PRESSO | -0.04 | 0.04 | -0.90 | 0.36 | 6 SNP |
|  | SCZ | Outlier-corrected MR-PRESSO | -0.03 | 0.03 | -1.08 | 0.28 | - |
| LDL-C | MDD | MR-PRESSO | 0.06 | 0.02 | 2.27 | 0.026 | **-** |
|  | Anxiety Disorder | MR-PRESSO | 0.13 | 0.05 | 2.64 | 0.01 | - |
|  | Panic Disorder | MR-PRESSO | -0.07 | 0.07 | -1.03 | 0.31 | - |
|  | PTSD | MR-PRESSO | -0.04 | 0.02 | -1.80 | 0.07 | - |
|  | AD | MR-PRESSO | 0.06 | 0.02 | 2.27 | 0.03 | - |
|  | SCZ | MR-PRESSO | 0.01 | 0.02 | 0.76 | 0.45 | - |
| TG | MDD | MR-PRESSO | 0.02 | 0.007 | 2.90 | 0.005 | **-** |
|  | Anxiety Disorder | MR-PRESSO | 0.13 | 0.05 | 2.75 | 0.007 | 1 SNP |
|  | Anxiety Disorder | Outlier-corrected MR-PRESSO | 0.13 | 0.05 | 2.97 | 0.004 | - |
|  | Panic Disorder | MR-PRESSO | -0.19 | 0.04 | -4.24 | <0.001 | - |
|  | PTSD | MR-PRESSO | -0.03 | 0.02 | -1.29 | 0.20 | **-** |
|  | AD | MR-PRESSO | 0.06 | 0.03 | 2.29 | 0.024 | 1 SNP |
|  | AD | Outlier-corrected MR-PRESSO | 0.05 | 0.02 | 2.97 | 0.004 | - |
|  | SCZ | MR-PRESSO | 0.04 | 0.02 | 1.74 | 0.08 | 2 SNP |
|  | SCZ | Outlier-corrected MR-PRESSO | 0.04 | 0.02 | 2.37 | 0.02 | **-** |

Abbreviations: MR, Mendelian randomization; TC, Total cholesterol; HDL-C, High density lipoprotein cholesterol; LDL-C, Low density lipoprotein cholesterol; TG, Triglycerides; MDD, Major Depressive Disorder; PTSD, Post-Traumatic Stress Disorder; AD, Alzheimer's Disease; SCZ, Schizophrenia; SD, standard deviation; SNP, single nucleotide polymorphism.

| Exposure | | Outcome |  | IVW | |  |  | Weighted median | |  |  | MR Egger | |  |
| --- | --- | --- | --- | --- | --- | --- | --- | --- | --- | --- | --- | --- | --- | --- |
|  |  | | OR (95% CI) | | P value | | OR (95% CI) | | P value | | OR (95% CI) | | P value | |
| MDD | TC | | 1.02(0.95-1.09) | | 0.62 | | 0.97(0.88-1.06) | | 0.51 | | 1.12(0.83-1.50) | | 0.45 | |
|  | HDL-C | | 1.00(0.94-1.07) | | 0.96 | | 0.97(0.88-1.06) | | 0.46 | | 1.03(0.76-1.38) | | 0.87 | |
|  | LDL-C | | 1.02(0.95-1.08) | | 0.64 | | 0.98(0.89-1.08) | | 0.70 | | 1.05(0.80-1.38) | | 0.70 | |
|  | TG | | **1.10(1.02-1.18)** | | **0.01** | | **1.10(1.01-1.21)** | | **0.036** | | **1.46(1.07-1.99)** | | **0.02** | |
| AD | TC | | 0.99(0.92-1.07) | | 0.81 | | 0.97(0.89-1.06) | | 0.50 | | 0.75(0.24-2.31) | | 0.70 | |
|  | HDL-C | | 0.95(0.74-1.22) | | 0.69 | | 1.05(0.93-1.20) | | 0.38 | | 0.07(0.02-0.21) | | 0.13 | |
|  | LDL-C | | 1.03(0.92-1.16) | | 0.53 | | 1.08(0.98-1.19) | | 0.14 | | 2.25(0.42-12.3) | | 0.52 | |
|  | TG | | 1.03(0.90-1.17) | | 0.67 | | 1.02(0.91-1.14) | | 0.74 | | 3.70(1.30-10.5) | | 0.25 | |
| SCZ | TC | | 1.00(0.97-1.03) | | 0.98 | | 1.00(0.98-1.03) | | 0.63 | | 1.08(0.91-1.28) | | 0.39 | |
|  | HDL-C | | 1.03(0.99-1.06) | | 0.096 | | 1.01(0.99-1.04) | | 0.28 | | 1.07(0.88-1.29) | | 0.51 | |
|  | LDL-C | | 0.99(0.96-1.01) | | 0.40 | | 1.00(0.97-1.03) | | 0.94 | | 1.01(0.87-1.18) | | 0.87 | |
|  | TG | | 0.99(0.96-1.02) | | 0.41 | | 1.00(0.98-1.03) | | 0.75 | | 1.04(0.89-1.22) | | 0.63 | |

**Supplementary Table 5.** MR Results of MDD/AD/SCZ on Risk of Blood lipids

Abbreviations: TC, Total cholesterol; HDL-C, High density lipoprotein cholesterol; LDL-C, Low density lipoprotein cholesterol; TG, Triglycerides; MDD, Major Depressive Disorder; AD, Alzheimer's Disease; SCZ, Schizophrenia; IVW, inverse-variance weighted MR; MR, Mendelian randomization; OR, odds ratio.

Associations of P < 0.05 are shown in bold.

**Supplementary Fig 1**. Venn plot of instrument variables of 4 fractions of blood lipid
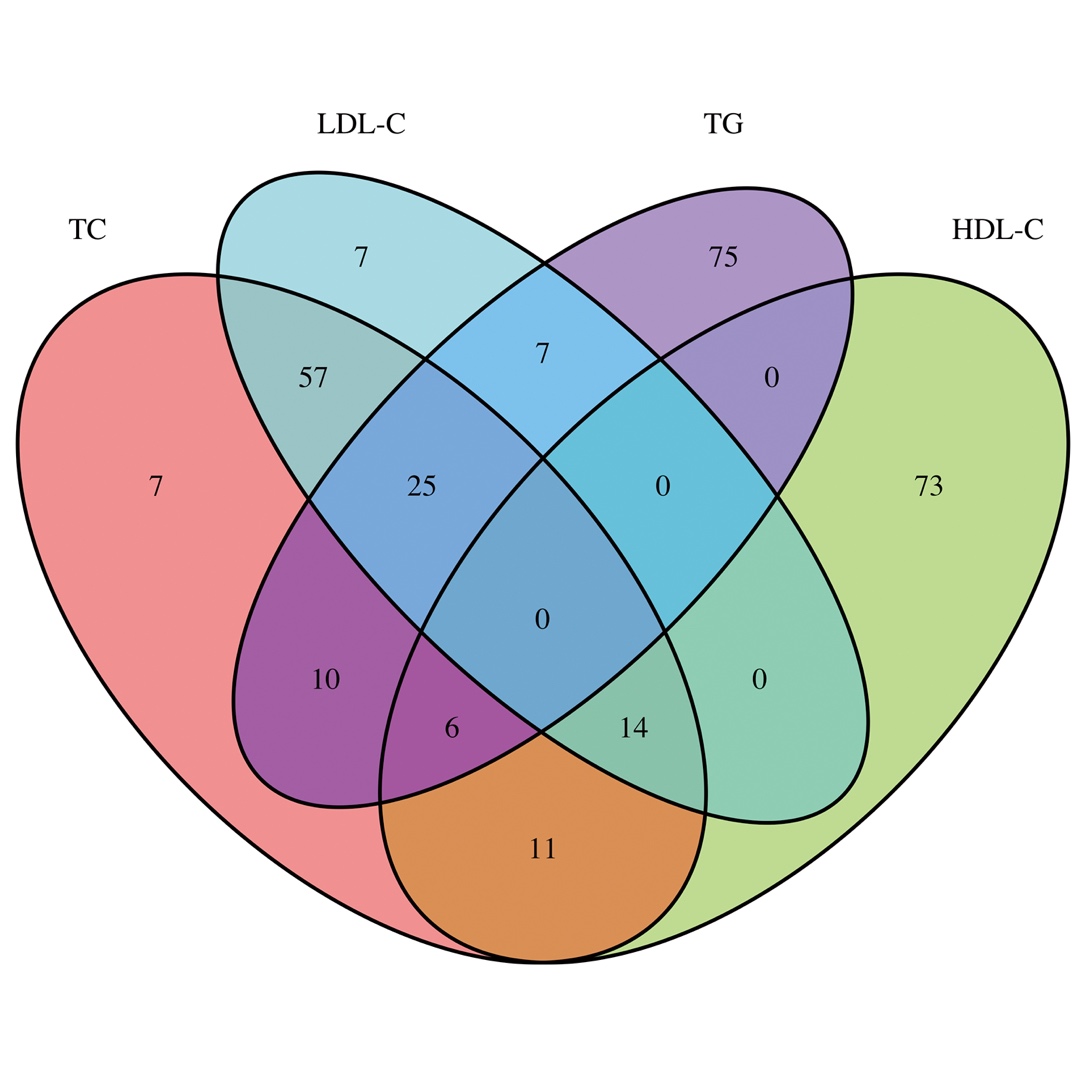


Abbreviations: TC, Total cholesterol; HDL-C, High density lipoprotein cholesterol; LDL-C, Low density lipoprotein cholesterol; TG, Triglycerides;
